# Examining epigenetic aging in the post-mortem brain in attention deficit hyperactivity disorder

**DOI:** 10.3389/fgene.2024.1480761

**Published:** 2024-10-08

**Authors:** Gauri G. Shastri, Gustavo Sudre, Kwangmi Ahn, Benjamin Jung, Bhaskar Kolachana, Pavan K. Auluck, Laura Elnitski, Philip Shaw

**Affiliations:** ^1^ Social and Behavioral Research Branch, National Human Genome Research Institute, NIH, Bethesda, MD, United States; ^2^ Zucker Hillside Hospital, Northwell Health, New York, United States; ^3^ Human Brain Collection Core, National Institute of Mental Health, NIH, Bethesda, MD, United States; ^4^ Translational and Functional Genomics Branch, National Human Genome Research Institute, NIH, Bethesda, MD, United States; ^5^ Pears Maudsley Center for Children and Young People, King’s College, Institute of Psychiatry, Psychology and Neuroscience, London, United Kingdom

**Keywords:** epigenetic age, ADHD (attention deficit and hyperactivity disorder), epigenetic clock, DNA methylation, biological age, postmortem brain

## Abstract

Mathematical algorithms known as “epigenetic clocks” use methylation values at a set of CpG sites to estimate the biological age of an individual in a tissue-specific manner. These clocks have demonstrated both acceleration and delays in epigenetic aging in multiple neuropsychiatric conditions, including schizophrenia and neurodevelopmental disorders such as autism spectrum disorder. However, no study to date has examined epigenetic aging in ADHD despite its status as one of the most prevalent neurodevelopmental conditions, with 1 in 9 children having ever received an ADHD diagnosis in the US. Only a handful of studies have examined epigenetic age in brain tissue from neurodevelopmental conditions, with none focused on ADHD, despite the obvious relevance to pathogenesis. Thus, here we asked if post-mortem brain tissue in those with lifetime histories of ADHD would show accelerated or delayed epigenetic age, as has been found for other neurodevelopmental conditions. We applied four different epigenetic clocks to estimate epigenetic age in individuals with ADHD and unaffected controls from cortical (anterior cingulate cortex, N = 55) and striatal (caudate, N = 56) post-mortem brain tissue, as well as peripheral blood (N = 84) and saliva (N = 112). After determining which epigenetic clock performed best in each tissue, we asked if ADHD was associated with altered biological aging in corticostriatal brain and peripheral tissues. We found that a range of epigenetic clocks accurately predicted chronological age in all tissues. We also found that a diagnosis of ADHD was not significantly associated with differential epigenetic aging, neither for the postmortem ACC or caudate, nor for peripheral tissues. These findings held when accounting for comorbid psychiatric diagnoses, substance use, and stimulant medication. Thus, in this study of epigenetic clocks in ADHD, we find no evidence of altered epigenetic aging in corticostriatal brain regions nor in peripheral tissue. We consider reasons for this unexpected finding, including the limited sampling of brain regions, the age range of individuals studied, and the possibility that processes that accelerate epigenetic age may be counteracted by the developmental delay posited in some models of ADHD.

## 1 Introduction

Alterations of the intrinsic biological aging process have been shown to be a feature of neuropsychiatric disorders. A widely used method for estimating biological age is to examine variations in the methylation of CpG sites with age (Horvath and Raj, 2018). Such “epigenetic clocks” are mathematical algorithms that take a linear combination of methylation values at a set of specific CpG methylation sites to estimate age (Field et al., 2018). Given that changes in the methylome are tissue specific, multiple epigenetic clocks have been trained on different tissue types (Bergsma and Rogaeva, 2020; Field et al., 2018; Horvath, 2013; McEwen et al., 2020; Shireby et al., 2020; Zhang et al., 2019), with most studies using peripheral tissues such as whole blood and saliva to estimate epigenetic age (Shenk et al., 2021; Wolf et al., 2019; Han et al., 2018; Protsenko et al., 2021; McEwen et al., 2020). However, studies focusing on changes in epigenetic markers in brain tissues are particularly important to advance our understanding of neuropsychiatric disorders.

Prior studies using post-mortem brain tissue have demonstrated acceleration of brain epigenetic aging in major depressive disorder (Han et al., 2018), bipolar disorder (Fries et al., 2020), and substance use disorders (Rosen et al., 2018; Poisel et al., 2023; Zillich et al., 2024). Studies in schizophrenia found delayed epigenetic aging in the cerebellum and frontal cortex (McKinney et al., 2017; AuthorAnonymous, 2018; Voisey et al., 2017; Liu et al., 2023; Teeuw et al., 2021; Wu et al., 2021). Among neurodevelopmental conditions, one study has demonstrated accelerated aging in ASD restricted to the cerebellum of older adults (Liu et al., 2023). Here we ask if a similar phenomenon applies to another neurodevelopmental condition, attention-deficit hyperactivity disorder (ADHD), which is characterized by impairing, age-inappropriate symptoms of inattention, hyperactivity, and impulsivity (Faraone et al., 2015). A prior study found increased ADHD genetic burden as measured by polygenic score to be associated with advanced epigenetic age in blood (Arpawong et al., 2023), and another study applied BrainAGE, a machine learning algorithm trained on structural neuroimaging data, to demonstrate delayed age in children with ADHD (Kurth et al., 2022). Importantly, no prior study to date has examined epigenetic aging in post-mortem brain tissue in ADHD. We focus on the caudate and anterior cingulate cortex (ACC), as neuroimaging studies have demonstrated subtle changes to their structure and function in ADHD (Hoogman et al., 2019; Hoogman et al., 2017; Hart et al., 2013; Yap, Abdul Manan, and Sharip, 2021). Further, the caudate and ACC are key components of corticostriatal circuitry that support cognitive processes pertinent to ADHD such inhibitory processing (Hart et al., 2013). Finally, the ACC and caudate are representative of cortical and subcortical regions, respectively, that could potentially capture alterations related to ADHD trajectory with age (Shaw and Sudre, 2021).

What alterations to epigenetic aging might be found in the brain in ADHD? We consider three possibilities. First, ADHD may be tied to accelerated biological aging in the brain, analogous to the shifts seen in other neurodevelopmental conditions such as ASD (Liu et al., 2023; Teeuw et al., 2021). Such accelerated epigenetic aging has been previously tied to the cumulative stresses of living, for example, with a neurodevelopmental challenge such as ADHD (Zannas et al., 2015). Secondly, ADHD, which is sometimes held to stem from developmental immaturity or delays, could be tied to a younger biological age–an instance of epigenetic age deceleration. Evidence for developmental immaturity has come from *in vivo* neuroimaging studies, including neuroanatomic (Muetzel et al., 2018; Shaw et al., 2013), functional connectivity (Francx et al., 2015; Clerkin et al., 2013; Schulz et al., 2017), and electrophysiological indices (Michelini et al., 2016; Cheung et al., 2016; Michelini et al., 2019). Finally, different brain regions may show different epigenetic ages. This proposition rests upon a model that ties the onset of ADHD to changes in the prenatal and infantile deep structures, such as the caudate, whereas the course of the disorder into adolescence and beyond is linked to prefrontal cortical plasticity, such as in the anterior cingulate cortex (Halperin and Kurt, 2006; Shaw and Sudre, 2021; Schulz et al., 2017). This model would suggest that the caudate and ACC might have different epigenetic ages, aligned with the different cognitive and neural changes occurring in these regions in ADHD.

In this study, we first determined which epigenetic clock algorithm(s) most accurately predicted chronological age in individuals with no lifetime psychiatric disorders. Next, we use the best performing clocks to determine if a diagnosis of ADHD is associated with altered epigenetic age in the brain. Finally, we ask if our findings for brain tissue hold in peripheral tissues (blood, saliva) obtained from a different cohort of youth with ADHD.

## 2 Materials and methods

### 2.1 Participant selection for postmortem brain specimens

Postmortem brain tissue was obtained from three brain banks: the NIMH Human Brain Collection Core, the Maryland Brain Collection, and the University of Pittsburgh Brain Tissue Donation Program. Anterior cingulate cortex and/or caudate tissue was obtained from 26 individuals with ADHD and 33 individuals with no psychiatric diagnoses (see Table 1). For the NIMH HBCC and Maryland Brain Collection, postmortem diagnostic interviews were performed with at least one family member of the deceased by a trained psychiatric social worker using the Structured Clinical Interview for the DSM-IV (SCID) and Diagnostic Evaluation After Death (DEAD). Two psychiatrists then reviewed the case write-up and arrived at a consensus for a possible DSM diagnosis, and in the case of the Maryland Brain Collection, medical records as well as records from the medical examiner’s office all contributed to the diagnosis (Donati et al., 2008). The University of Pittsburgh program similarly conducts a postmortem interview with the family of the deceased using a structured interview for the DSM-5, and this information is then reviewed during a consensus conference of psychiatrists and psychologists along with any medical records and publicly available records to arrive at a DSM diagnosis (Department of PsychiatryUniversity of Pittsburgh, 2020). Unaffected controls had no psychiatric disorders during the consensus diagnostic process. Race data was not available for one donor, so ACC and caudate tissue from this individual was excluded from the linear regression analysis where race was a covariate.

**TABLE 1:** Demographics of sample cohorts by biospecimen type.

	ACC	Caudate	Blood	Saliva
Total Individuals^a^	55	56	84	112
Male N (%)	46 (84%)	44 (79%)	56 (67%)	74 (66%)
White N (%)	36 (65%)	36 (64%)	62 (74%)	81 (72%)
White, non-Hispanic			56 (67%)	76 (68%)
Black/African American			8 (9%)	11 (10%)
Asian			4 (5%)	5 (4%)
More than one race/other			10 (12%)	15 (13%)
ADHD Diagnosis N (%)	24 (44%)	23 (41%)	35 (42%)	55 (49%)
Age mean (SD: range)	21.7 (8.0: 6.7–38.8)	22 (8.0: 6.7–38.8)	11.2 (2.7: 5.4–17.7)	10.2 (2.9: 4.5–18.4)
Individuals with ADHD who had comorbid psychiatric diagnoses	9 (37%) (1 ASD, 6 MDD/dysthymia, 1 BPAD, 1 MDD + GAD + panic disorder)	8 (35%) (1 ASD, 6 MDD/dysthymia, 1 BPAD)	8 (23%) (7 ODD, 1 panic disorder)	12 (22%) (9 ODD, 2 GAD, 1 DMDD)
Substance use disorders	12 (50%)	11 (48%)		
Stimulant medication			26 (71%)	47 (85%)

^a^ 52 individuals shared between ACC, and caudate, and 17 individuals shared between blood and saliva cohorts. ASD, autism spectrum disorder; MDD, major depressive disorder; BPAD, bipolar affective disorder; GAD, generalized anxiety disorder; ODD, oppositional defiant disorder; DMDD, disruptive mood dysregulation disorder.

### 2.2 Participant selection for blood and saliva specimens

Blood and saliva samples were obtained from a separate cohort based at the NIH Intramural Research Program in a study approved by the NIH central IRB. Individuals with no psychiatric diagnoses were contrasted against those with a diagnosis of ADHD, as determined by the clinician-administered Diagnostic Interview for Children and Adolescents-Fourth Edition (DICA-IV) with the parent. For adults aged 18 or over, diagnoses were made using the Connors’ Adult ADHD Diagnostic Interview for DSM-IV (CAADID; Epstein et al., 2001) and Structured Clinical Interview for DSM-IV Axis I Disorders (SCID). The main exclusion criteria were full scale IQ less than 70, birth before 32 weeks gestation, neurological disorder impacting brain structure, and psychoses. All adults and parents provided written informed consent, and written assent was obtained from children under 18. After methylation quality control, the final cohort included saliva specimens from 112 participants and peripheral whole blood specimens from 84 participants (see Table 1). There were 17 individuals who provided both saliva and blood. All tissues were from unrelated individuals.

### 2.3 Methylation data processing

Anterior cingulate cortex and caudate tissues were dissected from coronal slabs, which were prepared and frozen at −80°C during autopsy. DNA extraction and bisulfite conversion was performed on bulk homogenates of the dissected tissues using the EZ DNA Methylation Kit (Zymo Research, Irvine, CA, United States). In whole blood, DNA extraction and bisulfite conversion was performed using the EZ Methylation-Gold Kit (Zymo Research), and saliva was collected using the Oragene DNA collection Kit (Genotek).

All biospecimens were processed using the Infinium HumanMethylationEPIC BeadChip (Illumina, San Diego, CA), performed by the Genomics Core at the National Human Genome Research Institute (NHGRI). Methylation data was separated based on tissue type and processed using the *minfi* (Aryee et al., 2014), *wateRmelon* (Pidsley et al., 2013), and *ChAMP* (Tian et al., 2017) packages in R. Quality control involved removal of samples with incorrect predicted sex based on methylation values, poor quality based on plots of the log median intensity of methylated and unmethylated signals, mean detection p-value greater than or equal to 0.05, and bisulfite conversion rate less than or equal to 80%. These steps resulted in the removal of 0 brain samples, 0 blood samples, and 24 saliva samples. Probes with a detection p-value greater than or equal to 0.01 in any sample or bead count <3 in at least 5% of samples were removed, and only autosomal probes were considered. Methylation values were normalized using the Noob normalization method and were not batch-corrected, both as suggested by the authors of the Horvath Online Age Calculator (https://dnamage.genetics.ucla.edu) (Horvath, 2013). The final beta matrices were 827,701 CpG probes x 56 caudate specimens, 827,891 CpG probes x 55 ACC specimens, 828,686 CpG probes x 84 blood specimens, and 827,891 CpG probes x 112 saliva specimens.

### 2.4 Epigenetic age estimation and method comparison

Four epigenetic clocks were included in this study: the Cortical Clock trained on brain tissue by Shireby et al (Shireby et al., 2020), the DNAm multi-tissue clock by Horvath et al (Horvath, 2013), a blood and saliva-derived clock by Zhang et al that utilizes two different methods for age estimation (Zhang et al., 2019), and the PedBE clock trained on pediatric buccal epithelial cells by McEwen et al (McEwen et al., 2020). These clocks were selected because they were trained on cohorts with age ranges and tissue types (post-mortem brain, blood, or saliva) most similar to our study samples. Beta values were submitted to the Horvath Online Age Calculator, and the “Advanced Analysis” option was selected for blood samples to obtain immune cell proportion estimates based on the Horvath and Houseman methods (Horvath, 2013; Houseman et al., 2012). The remaining epigenetic age estimates were obtained using publicly available code published by the respective clock authors. The most accurate clock was selected for each tissue type by finding the lowest mean absolute difference between epigenetic and chronological age in unaffected (non-ADHD) individuals.

### 2.5 Examination of diagnostic differences in epigenetic aging

We examined the difference between chronological and epigenetic age in ADHD individuals versus unaffected individuals in the two brain regions separately. In this comparison, positive values indicate advanced epigenetic age, while negative values indicate younger epigenetic age as compared to chronological age. All regression analyses adjusted for the following technical factors: plate on which samples were processed during the EPIC methylation array procedure (“sample group”) and array scanner used to survey methylation sites. Analysis of brain tissue included additional technical covariates (brain bank of origin, post-mortem interval), neuronal proportion estimated using the R package *CETYGO* (selecting the most accurate estimates based on the CETYGO “score”) (Vellame et al., 2023; Hannon et al., 2024), and whether the manner of death was by suicide versus all other modes of death. The ADHD and unaffected groups did not differ significantly in proportion of sex assigned at birth (see Table 1) but did differ significantly in proportion of those of different race/ethnicity, and we adjusted for both these demographic factors. Mean chronological age did not differ significantly between groups for the post-mortem analyses but was also included as a covariate as suggested by the Horvath tutorial.

We tested the hypothesis that different brain regions would show different epigenetic aging using a mixed model regression, first testing for an interaction between diagnosis and brain region and including a random intercept term for donor identity. Brain tissue analyses were repeated with a subset of individuals with no comorbid psychiatric diagnoses (N = 45 for ACC and N = 47 for caudate), and with a subset of individuals without documented substance use (N = 42 for ACC and N = 44 for caudate, see Table 1).

Analyses of peripheral tissue were conducted in a similar manner, adjusting for relevant technical variables (methylation array plate and scanner) as well as race/ethnicity, sex assigned at birth, and chronological age. In blood analyses, there was further adjustment for immune cells known to be influenced by chronological age. Per the Horvath Online Age Calculator tutorial (Horvath, 2013), the included cell types were naïve CD8+ T cells, CD8+CD28-CD45RA- T cells, plasmablasts, CD4+ T cells, natural killer cells, monocytes, and granulocytes (see Models S1, S2). Cell abundance measures were generated using the Horvath Online Age Calculator’s “Advanced Analysis” option. For robustness, peripheral sample analyses were repeated with stimulant use as a covariate and again with only individuals with no comorbid psychiatric diagnoses (N = 76 for blood and N = 100 for saliva).

## 3 Results

### 3.1 Comparison of epigenetic clock performance in the brain

**FIGURE 1:**
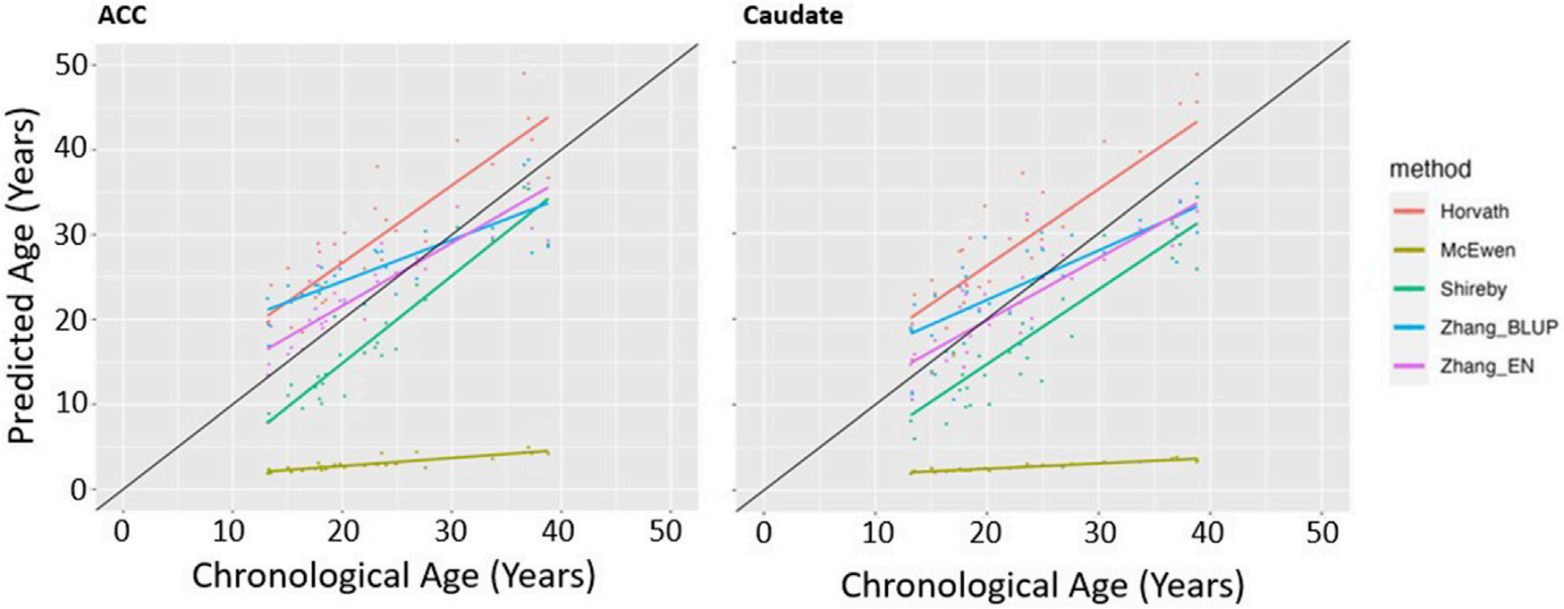
Epigenetic Clock Comparison: Plot of epigenetic versus chronological age for ACC and Caudate samples from unaffected (non-ADHD) individuals. Predicted ages are color-coded based on the clock used to generate the ages. The black line represents the line of perfect prediction (y = x) for reference.

We first determined which of the five epigenetic clock methods most accurately predicted chronological age in the brain regions, considering only the individuals with no lifetime history of psychiatric disorders. We found that the Zhang et al. elastic net method produced the lowest mean absolute age difference between predicted and actual age in both the ACC [absolute mean 2.6 years (SD 2.35 years)] and caudate [absolute mean 3.36 years (SD 2.26 years)]—see Table 2, Figure 1. For the caudate, this clock provided estimates of epigenetic age that were a mean of 0.97 years (SD 3.97) less than the chronological age; for the ACC, the epigenetic age was a mean of 1.00 years (SD 3.39 years) more than chronological age. The performance of the other clocks is shown in Table 2.

**TABLE 2:** Epigenetic clock comparison in years based on unaffected (Non-ADHD) individuals.

	ACC (N = 31)	Caudate (N = 33)	Blood (N = 49)	Saliva (N = 57)
	Abs age diff mean (SD)	Abs age diff mean (SD)	Abs age diff mean (SD)	Abs age diff mean (SD)
Horvath clock	6.62 (3.62)	6.67 (3.29)	**2.02** **(1.52)**	**1.31** **(1.12)**
McEwen clock	19.41 (7.02)	20.24 (7.53)	4.00 (2.34)	1.45 (1.16)
Shireby clock	5.18 (2.33)	5.66 (3.51)	7.15 (2.88)	14.21 (3.42)
Zhang clock: EN method	**2.60** **(2.35)**	**3.36** **(2.26)**	9.11 (2.72)	9.01 (2.99)
Zhang clock: BLUP method	5.08 (2.74)	4.61 (2.70)	5.73 (1.99)	6.36 (2.54)

Abs age diff = Absolute value of (Epigenetic Age–Chronological Age) in years.

Bolded values indicate the best performing algorithm.

### 3.2 Is ADHD associated with altered brain epigenetic age?

**FIGURE 2:**
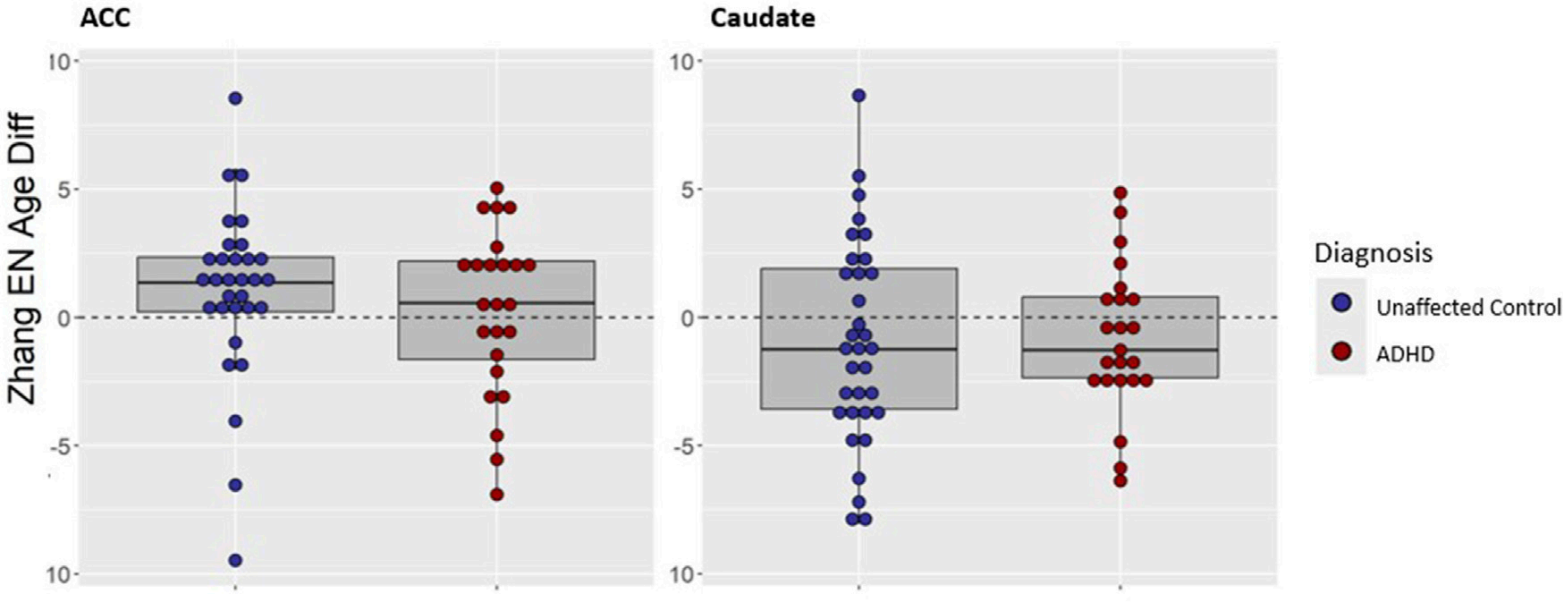
Comparison of age difference (epigenetic age–chronological age) in participants with ADHD versus unaffected volunteers in post-mortem ACC and caudate using the Zhang EN clock. Positive age difference indicates advanced epigenetic age.

As is illustrated in Figure 2, there was no diagnostic effect on the difference between epigenetic and chronological age for the caudate (t = −0.13, *p* = 0.90). Among those with ADHD, epigenetic age slightly underestimated chronological age in the caudate (mean −0.85, SD 2.85 years), similar to the underestimation observed in the unaffected group as noted above (mean −0.97, SD 3.97 years). For the ACC, epigenetic age among those with ADHD was again close to chronological age (mean 0.26, SD 3.22 years), and this did not differ significantly (t = 0.83, *p* = 0.41), from estimates for the unaffected group (mean 1.00, SD 3.39 years).

We next asked if there were differences in epigenetic aging between the two brain regions using mixed model linear regression, adjusting for covariates as described in the methods. We found no significant interaction between brain region and diagnosis in association with the age prediction error (beta = 0.60, t = 0.65, *p* = 0.52).

All findings held for analyses confined to those with no comorbid psychiatric diagnosis and when we excluded those with known substance use (Supplementary Table S1)

### 3.3 Association between ADHD and alterations in epigenetic age in peripheral tissues

**FIGURE 3:**
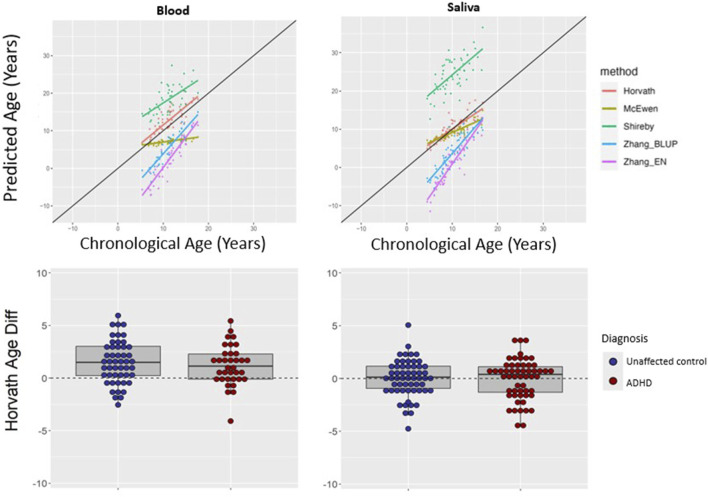
Epigenetic clock comparison and comparison of age prediction accuracy in peripheral tissues. Top two panels show epigenetic versus chronological age for blood (left) and saliva (right) samples from unaffected (non-ADHD) individuals. The black line represents the line of perfect prediction (y = x) for reference. The bottom two panels show the comparison of age difference (epigenetic age–chronological age) in participants with ADHD versus unaffected volunteers in blood (left) and saliva (right) using the Horvath multi-tissues clock. Positive age difference indicates advanced epigenetic age.

We asked if a similar pattern of results would be found for peripheral tissues. The Horvath multi-tissue clock performed best in predicting epigenetic age in unaffected individuals for both blood (mean absolute age difference 2.02 years [SD 1.52]) and saliva (mean absolute age difference 1.31 years [SD 1.12])—see Figure 3, Table 2.

Using the Horvath clock, for those with ADHD, epigenetic age predicted chronological age well in saliva (mean difference −0.05, SD 1.90 years). This did not differ significantly from the unaffected group (mean difference 0.02 years, SD 1.74, t = 0.22, *p* = 0.82). In blood from ADHD individuals, epigenetic age was a little over a year greater than chronological age (mean difference 1.20, SD 1.90 years), which did not significantly differ from estimates in the unaffected individuals (mean difference 1.51 years, SD 2.03, t = 0.73, *p* = 0.47), indicating a similar overestimation–see Figure 3. Findings held when stimulant use was included as a covariate, and when analyses were restricted to those without comorbid psychiatric diagnoses—see Supplementary Table S2.

## 4 Discussion

This is the first study to examine epigenetic aging in the brain of those with lifetime histories of ADHD. There were three central findings. First, we find that epigenetic clocks trained on peripheral tissue performed as well as, if not better than, clocks trained on brain tissue in estimating brain epigenetic age. Second, we find no evidence of significantly altered epigenetic aging in either the post-mortem anterior cingulate cortex or caudate of individuals with lifetime histories of ADHD. Finally, there are no ADHD-related differences in epigenetic aging in peripheral blood or saliva obtained from a separate cohort of individuals.

This study reinforces the validity of epigenetic clock algorithms in predicting chronological age across tissue types. It is noteworthy that the Zhang elastic-net clock most closely predicted chronological age in brain tissue from unaffected individuals, as this clock was initially trained on blood and saliva-derived methylation data. This suggests some degree of similarity between peripheral methylation changes and those found in the brain. This finding is consistent with a study by Cabrera-Mendoza et al. (Cabrera-Mendoza et al., 2023) which found significant positive correlations between blood and brain epigenetic age in healthy controls across multiple epigenetic clocks. Another consideration is differences in neuropathological load, as the age range of our post-mortem cohort is younger on average than the training cohort of the Cortical Clock by Shireby et al. Prior work has demonstrated that neurodegenerative pathology including neuritic plaques and amyloid load is associated with advanced epigenetic age (Levine et al., 2015). While the Cortical Clock excluded individuals with a known history of Alzheimer’s disease or other major neurological phenotypes, such histological findings can exist without clinical diagnosis and may skew the model. Finally, the PedBE clock by McEwen et al. performed the poorest in predicting chronological age in unaffected controls. This was also the only clock of those selected in this study to be trained exclusively on buccal epithelial cells from a pediatric cohort, potentially limiting its generalizability.

We found no evidence for altered epigenetic aging in ADHD, contrary to other neurodevelopmental conditions (Liu et al., 2023; Wu et al., 2021). There are several possible explanations. First, we examined only the caudate and ACC, while shifts in epigenetic age may lie in other brain regions. Such regional specificity in brain epigenetic patterns has been reported for other disorders. For example, Liu et al. (2023) demonstrated advanced epigenetic age in ASD in post-mortem cerebellar tissue of adults older than 45 years, but not in the prefrontal cortex or temporal cortex. The same study also found delayed epigenetic age in the post-mortem cerebellar tissue from adults with a diagnosis of schizophrenia aged 50–70 years, but not in the striatum or hippocampus. Secondly, there may be developmental effects. For example, studies suggest that there is delayed epigenetic aging in the frontal cortex among those with schizophrenia aged between 20 and 39 years, but not for those aged 60–90 years, though it is unclear whether neurodegenerative histopathology was fully accounted for in this work (Wu et al., 2021). Thus, it is possible that we might have found altered epigenetic aging if we had examined younger or older donors. Third, we used whole brain tissue homogenate, and methylation changes may be cell-specific. For example, several studies find accelerated epigenetic age to correlate with the proportion of non-neuronal cells, including oligodendrocytes and microglia, in the brain tissue samples used (Shireby et al., 2020; Stevenson et al., 2022; Murthy et al., 2023). However, we found no association between neuronal proportion and epigenetic age prediction accuracy in the present study. A final consideration is that ADHD may combine components of accelerated epigenetic aging, through the stress of living with a mental health challenge, with components of delayed epigenetic aging due to developmental immaturity—with these antagonistic processes “cancelling” one another out. Related to this possibility is the “last in, first out” hypothesis, developed based on P3 event-related potential data in ADHD, which proposes that the same brain regions with delayed maturation will also develop accelerated degeneration over time (Kakuszi et al., 2020).

There are several limitations to consider. First, the study’s biospecimens were obtained from different cohorts with no overlap between postmortem brain donors (ACC, caudate) and peripheral sample donors (blood, saliva). Our study samples were derived from a majority of white, non-Hispanic males, limiting the generalizability of our results. Additionally, the ADHD cohort included individuals with other comorbid psychiatric conditions as well as patients taking psychotropic medication. However, previous findings held when post-mortem brain analysis was repeated with a subset of individuals with no comorbid psychiatric illness, and again with the subset of individuals with no known substance use. Similarly, peripheral findings remained consistent when accounting for stimulant use or restricted to individuals with no comorbid psychiatric diagnoses.

In summary, we find no evidence of altered epigenetic aging tied to a diagnosis of ADHD in post-mortem ACC or caudate tissues, nor in peripheral blood or saliva. Future work might look at cell specific change, other brain regions, and a wider age range of individuals.

## Data Availability

The data presented in the study are deposited in the NIMH Data Archive, Collection 3151, Experiment 2443: https://nda.nih.gov/edit_collection.html?id=3151.
