# Supplementary material for: Examining epigenetic aging in the post-mortem brain in attention deficit hyperactivity disorder
Source: Front Genet. 2024 Oct 8;15:1480761. doi: 10.3389/fgene.2024.1480761 (PMC11493619; doi:10.3389/fgene.2024.1480761)
Supplement: Supplementary file 1 [file Table1.DOCX]

Supplementary Material

# Supplementary Equations

Model S1:

$$Blood Age Diff \sim Diagnosis+Chronological Age+CD8 naive+CD8pCD28nCD45RAn+PlasmaBlast+ CD4T+NK+Mono+Gran+Sex+Race+Sample Group+Scanner$$

Model S2:

$$Saliva Age Diff \sim Diagnosis+Chronological Age+Sex+Race+Sample Group+Scanner$$

# Supplementary Table

**Table S1. Epigenetic Age Comparison in ACC and Caudate of those with No Comorbid Psychiatric Diagnoses and Those with No Comorbid Substance Use.**

|  | **ACC Age Difference**  (Epigenetic Age – Chronological Age) | | | | **Caudate Age Difference**  (Epigenetic Age – Chronological Age) | | | |
| --- | --- | --- | --- | --- | --- | --- | --- | --- |
|  | Age Diff* Mean (SD) | *beta* | *t* | *p* | Age Diff Mean (SD) | *beta* | *t* | *p* |
| No Comorbid Psychiatric Diagnosis | ADHD:  0.24 (2.66)  Unaffected:  0.96 (3.44) | -0.69 | -0.68 | 0.50 | ADHD:  -0.74 (3.38)  Unaffected:  -0.88 (4.00) | 0.67 | 0.59 | 0.56 |
| No Substance Use | ADHD:  1.61 (2.09)  Unaffected:  1.00 (3.39) | 0.14 | 0.14 | 0.89 | ADHD:  0.16 (2.31)  Unaffected:  -0.97 (4.00) | -0.40 | -0.31 | 0.76 |

*Age difference (epigenetic age – chronological age) is in years.

**Table S2. Impact of Diagnosis on the Difference between Epigenetic and Chronological Age in Blood and Saliva.** Findings did not change when stimulant use was included as a covariate (for ADHD diagnosis, beta = 0.22, t=0.37, p = 0.72 for blood; beta = -0.07, t=-0.16, p = 0.87 for saliva) and when samples were restricted to those with no comorbid psychiatric disorders (beta = -0.03, t=-0.05, p = 0.96 for blood; beta = 0.38, t=1.11, p = 0.27 for saliva).

|  | **Blood DNAm Age Diff** | | | **Saliva DNAm Age Diff** | | |
| --- | --- | --- | --- | --- | --- | --- |
| *Predictors* | *beta* | *t* | *p* | *beta* | *t* | *p* |
| Intercept | -5.43 | -0.73 | 0.466 | 3.39 | 3.17 | **0.002** |
| Diagnosis (ADHD) | 0.16 | 0.32 | 0.749 | 0.13 | 0.39 | 0.700 |
| Chronological Age | -0.02 | -0.21 | 0.831 | -0.30 | -4.79 | **<0.001** |
| CD8 naive | 0.00 | 0.27 | 0.785 |  |  |  |
| CD8pCD28nCD45RAn | 0.14 | 1.33 | 0.189 |  |  |  |
| PlasmaBlast | 0.03 | 0.01 | 0.990 |  |  |  |
| CD4T | 4.80 | 0.45 | 0.654 |  |  |  |
| NK | 3.32 | 0.45 | 0.652 |  |  |  |
| Mono | 3.46 | 0.23 | 0.818 |  |  |  |
| Gran | 3.78 | 0.57 | 0.570 |  |  |  |
| Sex (Female) | -0.31 | -0.64 | 0.524 | -0.19 | -0.53 | 0.597 |
| Race | -1.00 | -1.96 | 0.055 | -0.52 | -1.47 | 0.144 |
| Sample Group 2 | 2.48 | 2.51 | **0.015** | 0.69 | 0.81 | 0.421 |
| Sample Group 3 | 0.83 | 0.89 | 0.377 | 0.06 | 0.07 | 0.947 |
| Sample Group 4 | -0.19 | -0.20 | 0.840 | 0.29 | 0.32 | 0.751 |
| Sample Group 5 | -0.32 | -0.32 | 0.748 | -0.93 | -1.14 | 0.257 |
| Sample Group 6 | 1.01 | 0.96 | 0.340 | -0.20 | -0.23 | 0.817 |
| Sample Group 7 | 0.40 | 0.40 | 0.692 | -0.93 | -1.07 | 0.287 |
| Sample Group 8 | 2.05 | 1.83 | 0.072 | -0.51 | -0.56 | 0.580 |
| Sample Group 9 | 1.77 | 1.61 | 0.113 | -0.50 | -0.51 | 0.609 |
| Sample Group 10 | 3.60 | 2.74 | **0.008** | -0.30 | -0.33 | 0.742 |
| Scanner (N0345) | 0.60 | 1.22 | 0.226 | 0.34 | 0.99 | 0.326 |
| Observations | 84 | | | 112 | | |
| R^2^ / R^2^ adjusted | 0.328 / 0.100 | | | 0.257 / 0.150 | | |
